# Dicyanamide Bridged Cu(II)36-Metallacrown-6 Complex with 1,4,7-Triisopropyl-1,4,7-Triazacyclononane and Binding Properties with DNA

**DOI:** 10.3390/molecules23061269

**Published:** 2018-05-25

**Authors:** Yong-Sheng Yang, Li-Jun Liu, Hai-Yan Ju, Xiu-Ying Liu, Yu-Guang Li, Shi-Ping Yan

**Affiliations:** 1 Hubei Key Laboratory of Biomass Fibers and Eco-dyeing & Finishing, School of Chemistry and Engineering, Wuhan Textile University, 1 Textile Road, Wuhan 430073, China; eduliu@163.com (L.-J.L.); juliesky2001@163.com (H.-Y.J.); liuxiuying@wtu.edu.cn (X.-Y.L.); liyg2010@wtu.edu.cn (Y.-G.L.); 2 Department of Chemistry, Nankai University, Tianjin 300071, China; yansp@nankai.edu.cn

**Keywords:** metallacrown, 1,4,7-triazacyclononane, dicyanamide, DNA binding properties

## Abstract

A novel 36-metallacrown-6 complex [CuL(N(CN)_2_)(PF_6_)]_6_∙0.5H_2_O **1** was achieved using a tridendate ligand, 1,4,7-triisopropyl-1,4,7-triazacyclononane (L), and a flexible ligand, dicyanamide in MeOH. The μ_1,5_ bridging models of the dicyanamide ligand linked the macrocycle to form in a specific size with the chair conformation. The anion was important to form this 36-metallacrown-6 complex, as change was obtained with the larger anion BPh_4_^−^, binuclear copper compound **2**. The magnetic property indicates that slightly ferromagnetic interactions resulted from a superexchange mechanism. DNA binding properties were also studied. UV and fluorescence spectra showed that complex **1** could bind with DNA.

## 1. Introduction

The control of ligand–field and metal–metal interactions in coordination chemistry has great effects for the development of novel magnetic and biologically active molecules. To optimize the properties, it is helpful to predict the relative arrangement of the metal ions, their geometry, and their stoichiometry within the molecular polynuclear complexes, in order to control the electronic and magnetic exchange interactions. The metallacrown strategy has led to advances in relevant areas, such as molecular magnetism [1,2,3,4,5] and luminescent complexes for biological imaging [6,7,8]. Metallacrowns have also been investigated for their multinuclear structures and interesting molecular architecture [9,10,11,12,13,14,15,16]. Many single-molecule-magnets (SMMs) are constructed by metallacrown complexes, such as DyX_4_M 12-Metallacrown-4 [17], Gd_2_Mn_4_ [3], Mn_6_(Ishz)_6_ [18], etc. The size of the metallamacrocycles can be determined by the nature of the bridging ligands and metal ions involved, such as the coordination geometries of the metal ions, the length of a bridging group between the metal centers, or the number of repeating units. Proper strategies are required to control the nuclear number of metallacrowns and obtain the desired compounds.

Most reported metallacrowns have been prepared using hydroxamic acids and/or ketonoximic acids as constructing ligands [19,20]. Previously, we reported a hexanuclear metallamacrocycle Ni complex which was obtained by using the tridentate ligand 1,4,7-triisopropyl-1,4,7-triazacyclononane and the flexible ligand dicyanamide (dca) [21]. Herein, we report a nonplanar metallacrown copper compound: [CuL(N(CN)_2_)(PF_6_)]_6_∙0.5H_2_ (L = 1,4,7-triisopropyl-1,4,7-triazacyclononane), using tridentate ligand L and a long bridging ligand dicyanamide for the construction of metallacrowns. 

**Figure 1:**
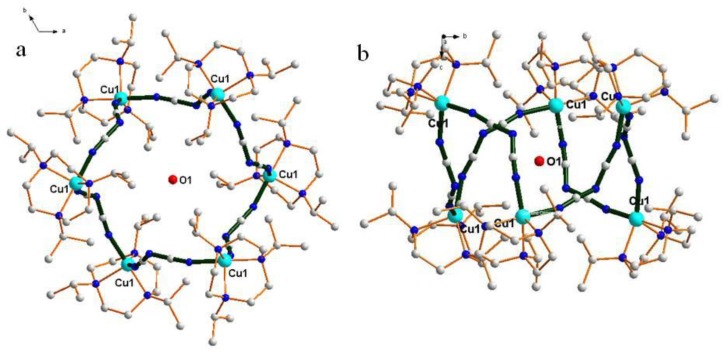
Diagram showing the perspective view of complex **1** with atom labels (**a**) from *c* axis and (**b**) from *a* axis.

**Figure 2:**
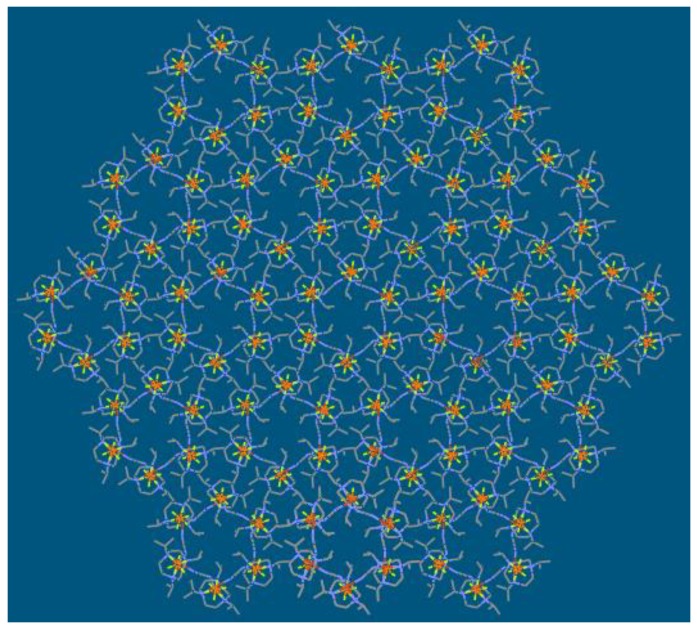
Stacking diagram of **1** along the *c* axis showing the regular array of hexahydric rings and the PF_6_^−^ anions.

Reaction of LCuCl_2_ species, NaN(CN)_2_ as bridging ligand, and KPF_6_ as anion in CH_3_OH solvent gave the hexanuclear complexes [22]. Single crystal X-ray analysis [23] of the compound illustrated that the compound is isomorphous and crystallized in the rhombohedral group $R_{\bar{3}}$. The crystal data and structure refinement are summarized in Table 1, and selected bond lengths (Å) and angles (°) are summarized in Table 2. Figure 1 gives a perspective view of compound **1**. The structure presents a regular hexagon from the *c* axis (Figure 1a). The copper atom was five coordinated with three N atoms from the L ligand and two N atoms from N(CN)_2_. Actually, the six copper atoms adopted a chair confirmation from the *a* axis (Figure 1b), where three Cu atoms were in the same plane and the other three Cu atoms were in another plane. N(CN)_2_^−^ anions bridged LCu^2+^ species, which formed a hexahydric Cu ring adopting the chair conformation. Cu···Cu···Cu angles and the Cu···Cu distances were the same, with 72.95° and 7.70 Å, respectively. The angles of N-Cu-N in the ring were 79.17°, while the C-N-C angle in the N(CN)_2_^−^ anion was 121.8(5)°. The packing diagram shows a regular array of hexahydric Cu rings and PF_6_^−^ anions along the *c* axis, with a regular honeycomb-like structure (Figure 2). PF_6_^−^ looks like blooming flowers on the ipr_3_tacn rings, forming cycles with 12 PF_6_^−^ anions. These values for the angles of the geometry demonstrate that the formation of hexahydric rings in this reaction is strongly favored. 

**Figure 3:**
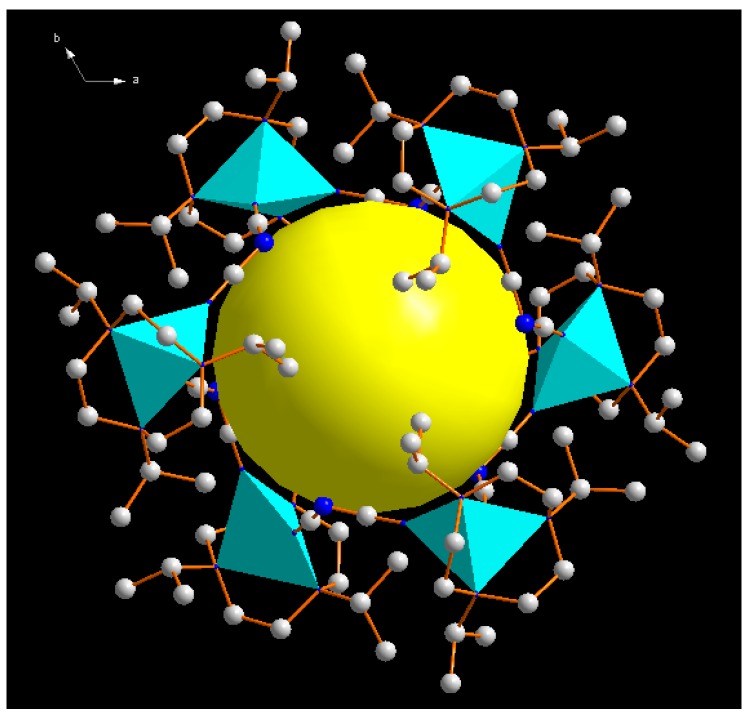
Molecular structure of the cyclic hexanuclear cation of **1** showing the cavity.

**Figure 4:**
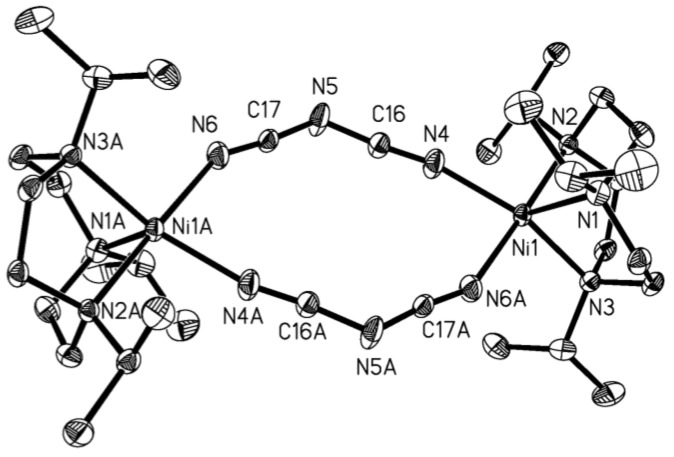
Perspective view of the binuclear NiII complex [LNi(N(CN)_2_)]_2_(BPh_4_)_2_ **2** with the atom-numbering scheme.

The distance from the center to the closest non-hydrogen atom of the ligand was 4.455 Å in **1**, thus the inner cavity had an estimated volume of ca. 80 Å^−3^ (Figure 3). Note that the anion was found to have a pronounced effect on the hexanuclear structure. When we changed the anion PF_6_^−^ to the larger anion BPh_4_^−^ with a similar nickel complex. A similar structure could not be obtained, but a binuclear Ni^II^ complex **2** was obtained (Figure 4) [24]. In complex **2**, the Ni ions were five-coordinated with the N(1), N(2), N(3) from L ligands and N(4), N(6) from dicyanamide ligands. Two Ni ions were bridged by dicyanamides, forming slightly distorted square-pyramidal geometry. The distances of Ni-N bonds were almost equal and amounted on average to 2.072 Å. The nearly planar dicyanamide utilized two of three possible N-donors to coordinate Ni ions, resulting in the formation of a twelve-membered, slightly puckered ring. The bond angle inside the ring, N(4)-Ni1-N(6), was 84.72(9)°. The intramolecular Ni···Ni separation was 7.386 Å.

**Figure 5:**
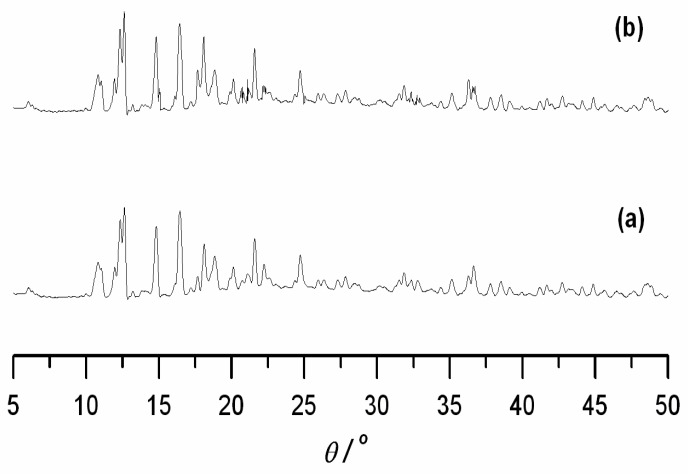
XRD patterns for compound **2**. (**a**) No adsorption of water molecules; (**b**) After adsorption of water molecules.

Although we performed the reactions in absolute CH_3_OH, water molecules were observed in compound **1** probably due to non-removed or osmotic water from surroundings. We speculate that the cavity of the two compounds might show abilities to absorb and store H_2_O molecules. The powder X-ray diffraction technique was used. The compound was immersed in water for 24 h, and the XRD patterns of the bibulous solid showed the main reflections remaining nearly identical with the pristine samples, which supported the notion that the crystal lattice of the compound would remain intact after absorbing the water molecules (Figure 5).

**Figure 6:**
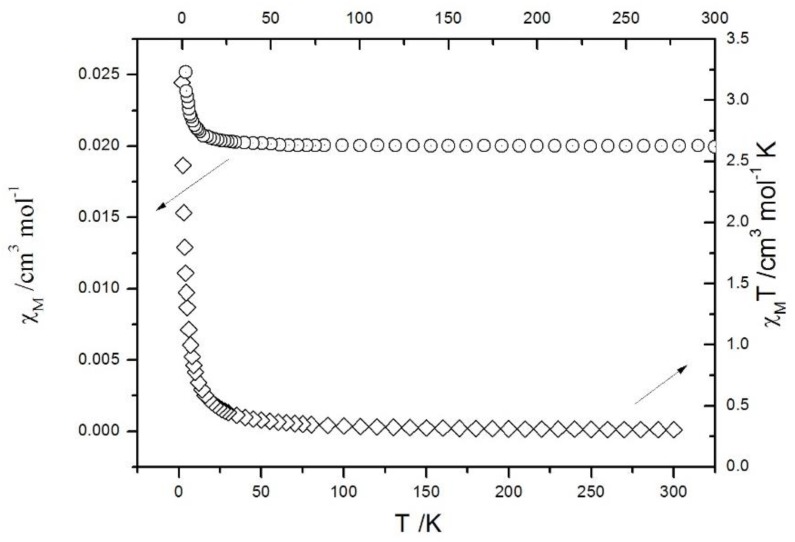
Plots of the magnetic susceptibility of complex **1**.

The magnetic susceptibilities were measured under a 1000 Oe applied magnetic field in the 2−300 K temperature range. As illustrated in Figure 6, the value of χ_m_T increased with decreasing temperature from 300 K to 2 K, showing the presence of an antiferromagnetic interaction between the Cu(II) ions. The slightly ferromagnetic interactions resulted from a superexchange mechanism of adjacent Cu(II) ions. With decreasing temperature, the χ_m_T increased slightly from 300 K to 24 K and then increased rapidly from 25 K to 2 K.

## 2. DNA Binding

**Figure 7:**
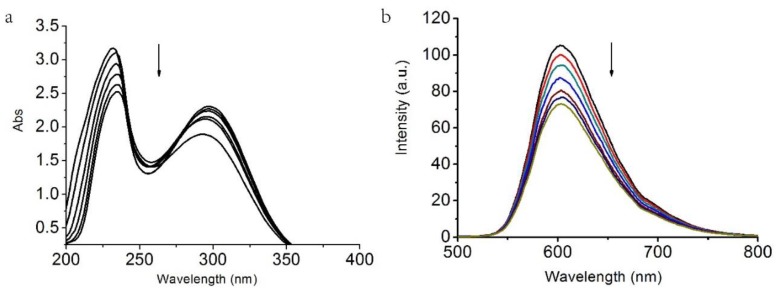
(**a**) Absorbtion spectra of complex **1** with increasing Calf-thymus DNA (CT-DNA). The arrow shows the absorbance changes with increasing DNA concentration. The concentration of the compound was 2.0 × 10^−5^ M, r = [CT-DNA]/[compound] = 0–0.6. (**b**) Fluorescence emission spectra (excited at 305 nm) of the CT-DNA-EB (ethidium bromide) system (2.5 μM EB, 100 μM CT-DNA) in the absence and presence of 1 × 10^–4^ M complex **1** with increasing concentrations of complex with 0 to 4 μM (from top to bottom).

In order to investigate whether DNA was the biological target of the compound, its interactions with Calf-thymus DNA (CT-DNA) were studied by UV-Vis and fluorescence spectroscopy. CT-DNA was prepared with Tris-HCl/NaCl buffer with pH of 7.5. The absorption spectra of compound **1** with absence and presence CT-DNA at various concentrations are shown in Figure 7a. The potential CT-DNA binding ability of complexes was studied by UV spectroscopy by following the intensity changes of the intraligand π–π* transition band at 232 nm and 298 nm. Upon the addition of an increasing amount of CT-DNA (from 10^−5^ to 10^−4^ M) to the complexes (10^−5^ M), 20% hypochromism and slight red shift (7–12 nm) were observed, indicating that interactions between the DNA phosphate groups and Cu cations, or Cu coordination to guanine bases, might have happened.

As a fluorescence spectral method, the relative binding of the complex to CT-DNA was studied though an ethidium bromide (EB)-bound CT-DNA solution with Tris-HCl/NaCl buffer (pH = 7.5). Fluorescence intensities at 610 nm were measured with various complex concentrations. Fluorescence titration spectra are shown in Figure 7b. The emission intensity showed a reduction with increasing concentration of complex **1**, suggesting that the compound can replace EB from CT-DNA and intercalate into the DNA double helix [25]. Ethidium bromide is an intercalator that gives a significant increase in fluorescence emission [26].

## 3. Conclusions

In summary, using ipr_3_tacn copper complex with dicyanamide as bridge ligand, we successfully synthesized and characterized a novel Cu(II)36-metallacrown-6 complex which contained regular hexacyclic metal rings adopting the chair conformation. Anion choice in preparation of the title compounds might play a significant role in the assembly of this metallacrown. The results in this article may provide an incremental advancement to the field of metallacrown chemistry.

## Figures and Tables

**Table 1:** Crystal data and structure refinement for **1** and **2**.

Compound	1	2
Empirical formula	C_17_H_33.17_CuF_6_N_6_O_0.08_P	C_82_H_106_Ni_2_B_2_N_12_
Formula weight	531.51	1398.83
Temperature	294(2) K	293(2) K
Wavelength	0.71073 Å	0.71073 Å
Crystal system, space group	Rhombohedral, *R*–3	Triclinic, *P*–1
Unit cell dimensions	*a* = 28.417(3) Å *α* = 90°	*a* = 10.2350(2) Å *α* = 74.4°
	*b* = 28.417(3) Å *β* = 90°	*b* = 10.8420(2) Å *β* = 88.8°
	*c* = 15.6781(19) Å *γ* = 120°	*c* = 18.055(3) Å *γ* = 78.5°
Volume	10964(2) Å^−3^	1890.2(6) Å^−3^
*Z*, Calculated density	18, 1.449 Mg m^−3^	1, 1.229 Mg m^−3^
Absorption coefficient	1.024 mm^−1^	0.550 mm^−1^
*F*(000)	4965	748
Crystal size	0.26 × 0.20 × 0.16 mm^3^	0.24 × 0.20 × 0.14 mm^3^
*θ* range for data collection	1.43–25.02°	1.99–26.40°
Limiting indices	−33 ≤ *h* ≤ 17, −33 ≤ *k* ≤ 33, −18 ≤ *l* ≤ 18	−12 ≤ *h* ≤ 12, −5 ≤ *k* ≤ 13, −22 ≤ *l* ≤ 22
Reflections collected/unique	18,801/4320 [*R*(int) = 0.0511]	10,744/7607 [*R*(int) = 0.0255]
Max. and min. transmission	1.000000 and 0.712842	1.000000 and 0.818876
Refinement method	Full-matrix least-squares on *F*^2^	Full-matrix least-squares on *F*^2^
Data/restraints/parameters	4320/54/287	7607/0/448
Goodness-of-fit on *F^2^*	1.066	1.006
Final *R* indices [*I* > *2θ*(*I*)]	*R*_1_ = 0.0421, *wR*_2_ = 0.1133	*R*_1_ = 0.0442, *wR*_2_ = 0.0860
*R* indices (all data)	*R*_1_ = 0.0691, *wR*_2_ = 0.1320	*R*_1_ = 0.0774, *wR*_2_ = 0.0990
Largest diff. peak and hole	0.558 and −0.362 e Å^−3^	0.326 and −0.290 e Å^−3^

**Table 2:** Selected bond lengths (Å) and angles (°) for **1** and **2**.

**1**			
Cu(1)-N(4)	1.992(3)	Cu(1)-N(2)	2.077(3)
Cu(1)-N(6)	2.001(4)	Cu(1)-N(3)	2.201(3)
Cu(1)-N(1)	2.070(3)		
N(4)-Cu(1)-N(6)	86.05(14)	N(1)-Cu(1)-N(2)	85.62(12)
N(4)-Cu(1)-N(1)	95.30(13)	N(4)-Cu(1)-N(3)	118.49(15)
N(6)-Cu(1)-N(1)	178.58(14)	N(6)-Cu(1)-N(3)	93.39(16)
N(4)-Cu(1)-N(2)	154.96(15)	N(1)-Cu(1)-N(3)	85.58(14)
N(6)-Cu(1)-N(2)	93.35(14)	N(2)-Cu(1)-N(3)	86.55(13)
**2**			
Ni(1)-N(4)	2.022(2)	Ni(1)-N(1)	2.065(2)
Ni(1)-N(6)	2.086(2)	Ni(1)-N(3)	2.0897(19)
Ni(1)-N(2)	2.0992(18)		
N(4)-Ni(1)-N(1)	117.56(9)	N(4)-Ni(1)-N(6)	84.72(9)
N(1)-Ni(1)-N(6)	93.79(9)	N(4)-Ni(1)-N(3)	154.51(9)
N(1)-Ni(1)-N(3)	87.87(8)	N(6)-Ni(1)-N(3)	95.90(8)
N(4)-Ni(1)-N(2)	93.74(8)	N(1)-Ni(1)-N(2)	87.51(7)
N(6)-Ni(1)-N(2)	178.33(8)	N(3)-Ni(1)-N(2)	85.19(7)

Symmetry operation is *P*–1 for **1** and *R*–3 for **2**.
